# Female multiple matings and male harassment and their effects on fitness of arrhenotokous *Thrips tabaci* (Thysanoptera: Thripidae)

**DOI:** 10.1007/s00265-015-1970-5

**Published:** 2015-07-18

**Authors:** Xiao-Wei Li, Jozsef Fail, Anthony M. Shelton

**Affiliations:** Department of Entomology, Cornell University, New York State Agricultural Experiment Station, Geneva, NY 14456 USA; Key Laboratory of Plant Protection Resources and Pest Management, Ministry of Education, Northwest A&F University, Yangling, Shaanxi 712100 China; Department of Entomology, Faculty of Horticultural Science, Corvinus University of Budapest, Budapest, 1118 Hungary

**Keywords:** Arrhenotokous *Thrips tabaci*, Female mating frequency, Male harassment, Fitness cost

## Abstract

Although it is generally assumed that one or a few matings are sufficient to maximize female fitness and that mating is generally assumed to be costly to females, multiple matings of females have been reported across a wide and taxonomically diverse set of animals. Here, we investigated female mating frequency and male harassment rate in arrhenotokous *Thrips tabaci*. In addition, the cost to females of mating, multiple matings, and male harassment to females was evaluated. We found that *T. tabaci* females mated multiple times during their lifetime and were subjected to a high rate of male harassment at all the ages we tested. Mating was costly to females in terms of reducing longevity and delaying the initiation of egg laying, although mating did not affect the survivorship and longevity of males. Furthermore, continual exposure to males also resulted in a fitness cost to mated females in terms of delayed egg production and reduced fecundity. Virgin females of arrhenotokous thrips produce only male progeny whereas mated females of arrhenotokous thrips produce males from unfertilized eggs and females from fertilized eggs. However, multiple matings did not allow females to fertilize a larger proportion of their eggs to increase the female offspring ratio. Our study demonstrates the conflicts between the occurrence of multiple matings and the cost of sexual activities. This raises questions about the evolution of multiple matings and polyandry in this species. Furthermore, these findings suggest that such phenomena may occur in other animal species and influence the evolution of their mating systems.

## Introduction

Mating frequencies of females vary considerably with different mating systems (Thornhill and Alcock 1983). It is generally assumed that a single or few matings may provide females with sufficient sperm to reach their reproductive potential (Walker 1980; Arnqvist and Nilsson 2000). In addition, for females, there are fitness costs associated with mating, including energetic and time costs for other activities (Daly 1978; Kotiaho et al. 1998b; Watson et al. 1998; Franklin et al. 2012), the risk of increased predation (Wing 1988; Kotiaho et al. 1998a; Magnhagen 1991), physical damage (Parker 1979; Leboeuf and Mesnick 1991; Crudgington and Siva-Jothy 2000), toxic seminal fluid (Chen 1984; Chapman et al. 1995), and immunity corruption (Rolff and Siva-Jothy 2002). These costs may decrease a female’s lifespan and egg production rate (Arnqvist and Nilsson 2000). Consequently, females usually favor a lower mating rate compared to males (Parker 1979) and are resistant or reluctant to re-mate (Kokko et al. 2003).

Despite the cost of mating, multiple matings of females, most often with different males but also with the same male, have been widely reported in diverse animals (Arnqvist and Nilsson 2000). Frequent multiple matings can be explained by the benefits females may gain from re-mating, including increased offspring production (Arnqvist and Nilsson 2000; Blanckenhorn et al. 2002; Ji et al. 2007; Gotoh and Tsuchiya 2008), increased genetic diversity of offspring (Jennions and Petrie 2000), and beneficial accessory substances transferred by males during mating (Thornhill 1976; Eberhard and Cordero 1995; Vahed 1998). However, multiple matings may be costly for individuals in terms of reducing fecundity (Sirot and Brockmann 2001; Johnson and Brockmann 2010; Oku 2010; Ronkainen et al. 2010) or longevity (Arnqvist and Nilsson 2000). These costs may be due to the act of mating itself and/or sexual harassment by males during multiple matings. Male harassment, defined as a male’s repeated attempts to mate (Cluttonbrock and Parker 1995), is reported to be costly to females in many animal species (Chilvers et al. 2005; Plath 2008; Gay et al. 2009; Rossi et al. 2010; Helinski and Harrington 2012).

Mating frequencies of females differ between species in the order Thysanoptera, a taxon of insects important in agriculture. Multiple matings have been reported in females from some tubuliferan species, either repeated matings with the same males (e.g., *Elaphrothrips tuberculatus* (Hood), *Hoplothrips pedicularius* (Haliday), and *H. karnyi* (Hood)) (Crespi 1986, 1988a, b) or multiple matings with different males (e.g., *Dunatothrips aneurae*Mound) (Gilbert and Simpson 2013). However, females of some gall-forming thrips mate once and refuse further matings (Varadarasan and Ananthakrishnan 1982). In *Frankliniella occidentalis* (Pergande), after an initial mating, females refused males for more than 5 days (Terry and Schneider 1993) while in *Echinothrips americanus*Morgan, the majority of females refused to re-mate during a 30-day period (Li et al. 2014b).

Onion thrips, *Thrips tabaci*Lindeman (Thysanoptera: Thripidae), is a serious global insect pest because of its direct feeding on many agricultural crops, its ability to transmit viruses, and its resistance to many insecticides (Diaz-Montano et al. 2011). As in other thysanopteran species, arrhenotokous *T. tabaci* is haplodiploid: virgin females only produce haploid male offspring while mated females can produce both diploid female offspring and haploid male offspring. Since mating is not a prerequisite of egg production in arrhenotokous *T. tabaci* (both virgin and mated females can produce eggs), arrhenotokous *T. tabaci* is a good model organism to investigate the cost of mating on fecundity and longevity. In addition, there is no report about female mating frequency and sexual harassment behavior in this species.

In this study, we investigated female mating frequencies and male harassment rate in arrhenotokous *T. tabaci*. In addition, the effect of mating on the fitness of female and male *T. tabaci* was investigated by comparing virgin and mated female and male *T. tabaci*. Last, the fitness costs to females by multiple matings and male harassment were investigated by comparing individually housed mated females and mated females exposed to males.

## Materials and methods

### Population maintenance and insect rearing

The arrhenotokous *T. tabaci* population used in this study was established in 2011 from adult *T. tabaci* collected from cabbage in a research field (GPS coordinates 42.873621, -77.029556) of Cornell University’s New York State Agricultural Experiment Station, Geneva, New York. The population was maintained on potted onion plants or cabbage heads in environmental growth chambers at 20 ± 1 °C, 60 ± 5 % relative humidity (RH), and a photoperiod of 16 L/8 D. Sequencing of a 706-bp COI fragment of several *T. tabaci* individuals identified one haplotype in our arrhenotokous colony (Li et al. 2014a) belonging to the leek-associated clade (Brunner et al. 2004), and it has been proposed that the leek-associated clade of *T. tabaci* is arrhenotokous (Toda and Murai 2007). To confirm arrhenotoky in the tested individuals, the following procedure was applied: 21 females randomly selected from the stock colony were isolated individually in 1.7-ml microcentrifuge tubes with cabbage head leaf disks (5 mm in diameter) serving as a food source and oviposition site, and their progeny was raised to adulthood also in isolation after egg hatching. F_1_ females and males produced by different mothers were coupled in order to avoid inbreeding. The F_2_ progeny was raised similarly, and adults of this generation were used in the tests. The female/male ratio in the F_2_ progeny was 77:23, which is about the expected 4:1 sex ratio in arrhenotokous thrips (Lewis 1973). The relatedness of isolated specimens in the F_1_ and F_2_ progenies was recorded, and F_2_ sisters produced by the same F_1_ female were allocated to all treatments as evenly as possible (with at least one sister in any tested F_2_ line allocated to the virgin female alone treatment). The exclusive male progeny of the F_2_ sisters in the virgin female alone treatment confirmed arrhenotoky in all tested individuals.

### Female mating frequencies and male harassment rate

Mating behavior of a virgin female paired with a single male (2–7 days old) was recorded using a video recorder (ZC105 Megapixel Camera, Zarbeco, NJ, USA) for 1 h at female ages of 2, 4, 6, 8, 10, 15, 20, 25, and 30 days. Two treatments were established: (1) a single virgin female (*n* = 20) paired with a single virgin male companion and (2) a single virgin female (*n* = 20) paired with a single mated male companion. A total of 20 replications was included in both mated and virgin male treatments, but insects accidentally killed were excluded when we calculated female mating frequency (2 in virgin male treatment and 1 in mated male treatment). On each recording day, males were removed after 1 h of recording, and females were left individually until the next recording day. During recording breaks, females were held in 1.7-ml microcentrifuge tubes with cabbage head leaf disks (5 mm in diameter) that were changed daily under the rearing conditions described above. Accumulated female mating frequencies in the two treatments (virgin female paired either with a mated or virgin male) during the first 10-day period and the entire 30-day period (which is close to the mean longevity of mated females) were calculated, respectively. Accumulated percentages of re-mated females over time in the two treatments were also calculated.

Male harassment rates (male harassment incidences per hour) in the two treatments at different female ages were also determined. We considered a male harassment incident when the male attempted mating, i.e., mounted the female’s back or twisted his abdomen sideways under the end of the female’s abdomen (Lewis 1973), but it was refused by the female. To investigate if male harassment rate is equal at different ages of a female, we calculated average male harassment rates in the two treatments during the first 10-day period and the entire 30-day period. The 10-day period was selected for statistical analysis because a significant drop was observed in harassment rates for *Echinothrips americanus* after 10 days of age of tested females (Li et al. 2014b) and 30 days is about the mean lifespan of mated *T. tabaci*.

### Effects of mating on the fitness of females and males

Newly emerged virgin females (*n* = 58) and mated females (*n* = 58, paired with a single male companion for 2 days) were individually confined in microcentrifuge tubes and reared at conditions as described previously. Leaf disks were changed at 12-h intervals until the beginning of oviposition. The preoviposition period was calculated as the time from adult emergence to the beginning of oviposition. When females began laying eggs, leaf disks were changed at 24-h intervals, and the number of eggs in the leaves was counted using the bottom light of a stereomicroscope (ZEISS, Stemi 2000, Carl Zeiss Microscopy, Jena, Germany). Oviposition period (i.e., the period between the first and the last egg laid, measured in days), longevity (i.e., the period between the emergence and the death of the adult, measured in days), lifetime fecundity (i.e., total number of eggs laid), and daily fecundity (lifetime fecundity divided by oviposition period) were calculated for each female. The survivorship (i.e., the proportion of individuals surviving to a particular age) of virgin and mated females was calculated as well.

Newly emerged virgin males (*n* = 36) and mated males (*n* = 38, paired with a female for 2 days) were reared under the same conditions as described above. Longevity and survivorship of virgin and mated males were determined.

### Effects of multiple matings and male harassment on fitness of females

To test the effect of multiple matings and male harassment on fitness of females, two treatments were established: (1) 1 mated female (*n* = 58, paired with a single male companion for 2 days) kept alone and (2) 1 female and 1 male kept together until the female’s death (*n* = 56). In the second treatment, in case of a male’s death before the female’s, a new male companion (2–7 days old) was added to replace the dead one so that each female was accompanied by a male during her lifetime. Thrips in the two treatments were confined in microcentrifuge tubes under the same conditions as described above. The preoviposition period, oviposition period, longevity, survivorship, lifetime fecundity, and daily fecundity were calculated. To determine the sex ratio of offspring from females in different treatments, eggs laid by females in the mated female kept alone and mated female with a male companion treatments were kept for analysis of sex ratios when the eggs developed into adults.

### Statistical analysis

All data analyses were performed in SPSS (v20, SPSS Inc, Chicago, IL, USA). Prior to analysis, data were checked for normality using nonparametric Kolmogorov-Smirnov and Shapiro-Wilk tests (*P* < 0.05) as well as studying skewness and kurtosis according to Tabachnick and Fidell (2007). The four response variables in the mating behaviour study (accumulated female mating frequencies in 10 and 30 days of age, average male harassment rates in 10 and 30 days age of females) were moderately correlated (Pearson’s correlation coefficients and their *p* values are listed in Table 1); therefore, a one-way multivariate analysis of variance (MANOVA) was conducted to test the hypothesis that there would be mean differences between the treatments (virgin male, mated male). The normality of the residuals was confirmed by their skewness and kurtosis because all of these absolute values were <1. Prior to conducting a series of follow-up *t* tests, the homogeneity of variance assumption was tested for all response variables. Mean harassment rates in every female age tested were calculated, and the treatments (virgin or mated male) were compared by using a *t* test. The accumulated percentage of females re-mating with a male (virgin or mated) was also calculated. Since the response variables (longevity, fecundity, daily fecundity, preoviposition, and oviposition period) in the female fitness study were also dependent ones (Pearson’s correlation coefficients and their *p* values are listed in Table 2), a MANOVA was conducted with treatment (virgin female, mated female kept alone, and mated female accompanied by a male) as a fixed factor and maternal grandmother (the female randomly selected from our stock colony) as a random factor. We calculated the partial Eta squared value to detect the effect sizes as well as the observed power which gives the probability of correct detection of significant differences. The normality of the residuals was confirmed by their skewness and kurtosis: all of these absolute values were <1. Prior to conducting a series of follow-up ANOVAs, the homogeneity of variance assumption was tested and confirmed for all five variables (longevity, fecundity, daily fecundity, preoviposition, and oviposition period). Post hoc comparisons were done using a Dunnett *t* test with the mated female kept alone treatment chosen as the reference. Because there was no variation in the last response variable (female ratio in progeny) in the virgin female treatment (100 % male progeny), this response variable was analyzed in a univariate GLM only at two treatment levels (and excluded from the MANOVA test) with treatment (mated female kept alone and mated female accompanied by a male) as a fixed factor and maternal grandmother as a random factor. Male longevity was also analyzed in a univariate GLM with treatment (virgin or mated) as a fixed factor and maternal grandmother as a random factor. To normalize distributions, the percentage data of the female ratio in progeny was arcsine transformed, male longevity was log transformed, and preoviposition period was inverse transformed prior to analysis, but untransformed means and their 95 % confidence intervals (CI) are presented for all variables. For survival analysis, a log-rank test was performed in the Kaplan-Meier survival analysis procedure to compare the survival distributions of female and male adults between different treatments (virgin and mated male; virgin female; mated female kept alone or with a male).

**Table 1:** Bivariate Pearson’s correlation matrix of male harassment rates and female mating frequencies

		Male harassment rate		Female mating frequency	
Male harassment rate		First 10 days	Entire 30 days	First 10 days	Entire 30 days
First 10 days	Pearson’s r	1	0.944	−0.565	−0.243
	P		0.001	0.001	0.147
Entire 30 days	Pearson’s r	0.944	1	−0.587	−0.290
	P	0.001		0.001	0.082
Female mating frequency					
First 10 days	Pearson’s *r*	−0.565	−0.587	1	0.652
	*P*	0.001	0.001		0.001
Entire 30 days	Pearson’s *r*	−0.243	−0.290	0.652	1
	*P*	0.147	0.082	0.001	

Number of tested individuals *N* = 37

**Table 2:** Bivariate Pearson’s correlation matrix of oviposition, longevity, fecundity, daily fecundity, and preoviposition period of *Thrips tabaci* females in 3 treatments (virgin, mated kept alone, mated with a male companion)

		Oviposition	Longevity	Fecundity	Daily fecundity	Preoviposition period^a^
Oviposition	Pearson’s *r*	1	0.946	0.832	0.008	0.251
	*P*		0.001	0.001	0.924	0.002
	*N*	149	149	149	149	149
Longevity	Pearson’s *r*	0.946	1	0.781	−0.108	0.012
	*P*	0.001		0.001	0.190	0.880
	*N*	149	160	160	149	149
Fecundity	Pearson’s *r*	0.832	0.781	1	0.477	0.351
	*P*	0.001	0.001		0.001	0.001
	*N*	149	160	160	149	149
Daily fecundity	Pearson’s *r*	0.008	−0.108	0.477	1	0.317
	*P*	0.924	0.190	0.001		0.001
	*N*	149	149	149	149	149
Preoviposition period^a^	Pearson’s *r*	0.251	0.012	0.351	0.317	1
	*P*	0.002	0.880	0.001	0.001	
	*N*	149	149	149	149	169

^a^Statistical analysis was carried out following inverse transformation

*N* number of replications

## Results

### Female mating frequency

**Fig. 1:**
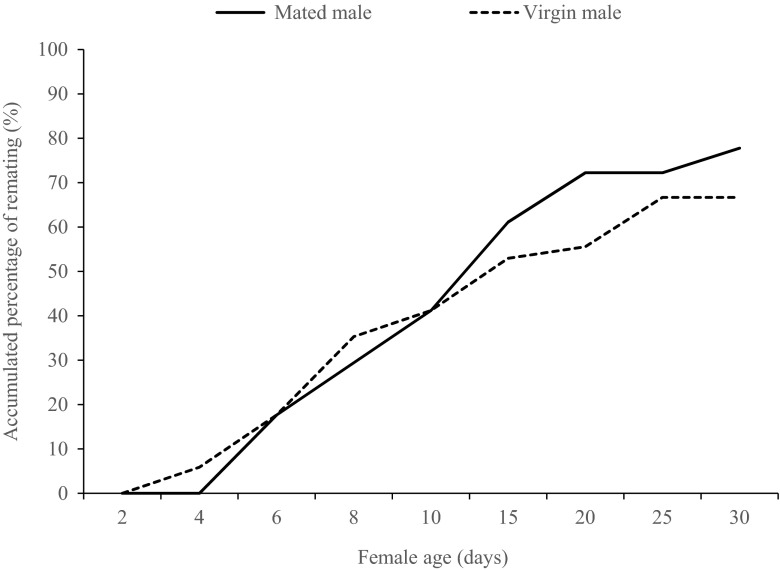
Accumulated percentage of re-mated females paired with a mated orvirgin male in *Thrips tabaci*

In both mated male and virgin male treatments, female re-mating behavior occurred. During the first 10 days, an equal proportion of the females (41 ± 0.6 %) re-mated with virgin and mated males (Fig. 1). During the entire 30-day period, 78 ± 0.6 % of females re-mated with mated males while 67 ± 0.7 % of females re-mated with virgin males (Fig. 1). The experience of the male did not have a significant model effect on female mating frequencies (Wilks’ Λ = 0.923; *F*(4, 32) = 0.665; *p* = 0.621), and there were no significant differences detected by the follow-up univariate tests in the accumulated mating frequency between females paired with mated and virgin males during the first 10 days (*F*(1, 35) = 0.586; *p* = 0.449) and the entire 30 days (*F*(1, 35) = 0.001; *p* = 0.971) period (Table 3).

**Table 3:** Accumulated female mating frequency and male harassment rate of a female paired with a single mated or virgin male during the first 10-day period and entire 30-day period

	Female mating frequency		Male harassment rate	
	First 10 days	Entire 30 days	First 10 days	Entire 30 days
With virgin male	1.44 ± 0.34 (18)	2.33 ± 0.68 (18)	21.6 ± 3.32 (18)	21.7 ± 2.70 (18)
With mated male	1.26 ± 0.33 (19)	2.32 ± 0.67 (19)	25.4 ± 3.24 (19)	24.6 ± 2.63 (19)
*P*	0.449	0.971	0.108	0.121

Estimated means (±95 % CI) calculated by a general linear model within a column were compared by *t* tests. Number of tested individuals is in brackets

### Male harassment rate

**Fig. 2:**
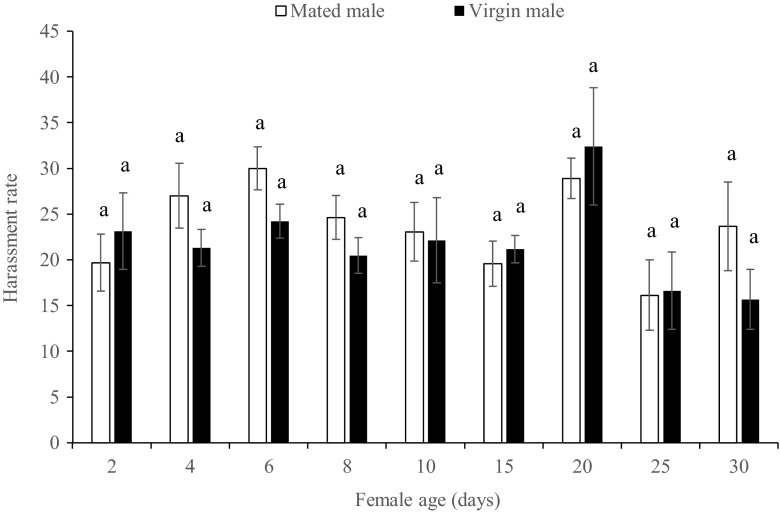
Harassment rate of mated and virgin males toward *Thrips tabaci* females at different ages. *Different letters* indicate significant difference (*t* test, *p* < 0.05)

Regardless of being paired with virgin or mated males, females at different ages suffered high rates of male harassment (Fig. 2). In general, there was no model effect of male harassment frequency (Wilks’ Λ = 0.923; *F*(4, 32) = 0.665; *p* = 0.621) between mated males and virgin males, and average male harassment rates were equal between females paired with mated or virgin males during the first 10-day (*F*(1, 35) = 2.713; *p* = 0.108) and the entire 30-day (*F*(1, 35) = 2.528; *p* = 0.121) period (Table 3). Subsequent *t* tests yielded no significant differences between virgin and mated males at any female age (Fig. 2).

### Effects of mating on the fitness of female and male adults

Female fitness was affected by treatment (Wilks’ Λ = 0.604; *F*(10, 244) = 6.999; *p* < 0.001; partial η^2^ = 0.223; observed power = 0.999) and maternal lineage (Wilks’ Λ = 0.314; *F*(100, 600) = 1.607; *p* < 0.001; partial η^2^ = 0.207; observed power = 0.999). A series of one-way ANOVA’s on each of the five dependent variables revealed a significant effect of maternal lineage on preoviposition period only (*F*(20, 126) = 3.463; *p* < 0.001; partial η^2^ = 0.355; observed power = 0.999). Maternal lineage had no effect on the other response variables.

The result of follow-up univariate tests on each of the five dependent variables in our MANOVA model revealed statistically insignificant treatment effect on daily fecundity (*F*(2, 126) = 2.151; *p* = 0.121), and statistically significant effect on longevity (*F*(2, 126) = 4.579; *p* = 0.012; partial η^2^ = 0.068; observed power = 0.768), preoviposition (*F*(2, 126) = 22.975; *p* < 0.001;partial η^2^ = 0.355; observed power = 0.999) and oviposition period (*F*(2, 126) = 11.512; *p* < 0.001; partial η^2^ = 0.154; observed power = 0.993), and fecundity (*F*(2, 126) = 9.597; *p* < 0.001; partial η^2^ = 0.132; observed power = 0.979).

**Fig. 3:**
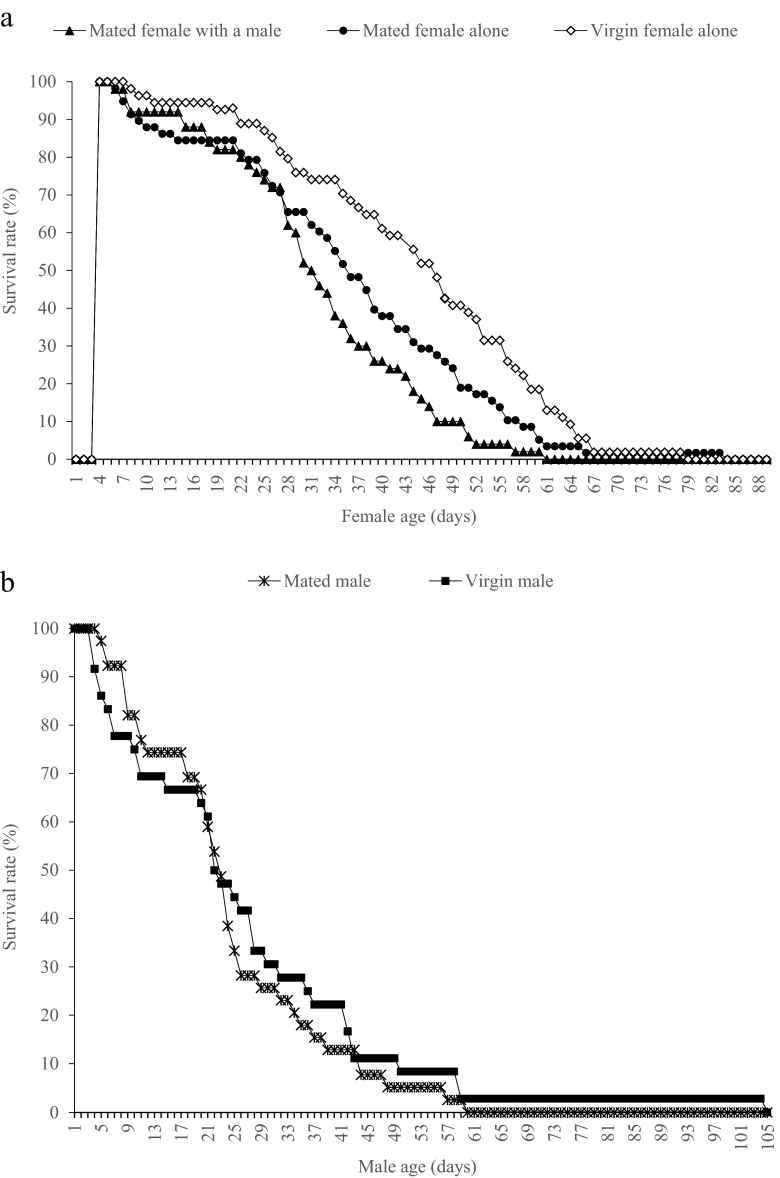
Survivorship of *Thrips tabaci* females and males in different treatments. **a** Females, **b** males

The results of the post hoc analyses are presented in Table 4. Compared to the reference treatment (mated female kept alone), virgin females had a significantly shorter preoviposition period. The oviposition period and longevity of virgin females were longer than that of mated females kept alone. There were no differences in the daily and lifetime fecundity between virgin females and mated females kept alone. Mating also affected the survivorship of females. Compared with virgin females, the survival rate of mated females was significantly lower (Fig. 3a; log-rank test: *χ*^2^_1_ = 5.30; *p* = 0.021).

**Table 4:** Female and male longevity, female preoviposition period, oviposition period, fecundity, and daily fecundity of virgin and mated *Thrips tabaci*

	Female longevity (days)	Male longevity^a^ (days)	Preoviposition period^a^ (days)	Oviposition period (days)	Fecundity(eggs/female)	Daily fecundity^*b*^(eggs/female/day)
Virgin	40.1 ± 4.64 (52)	27.8 ± 7.43 (36)	3.4 ± 1.16 (52)	35.2 ± 4.60 (52)	115.7 ± 18.2 (52)	3.5 ± 0.35 (52)
Mated	36.5 ± 4.33 (52)	23.8 ± 4.51 (38)	5.5 ± 1.09 (52)	29.8 ± 4.29 (52)	101.1 ± 17.0 (52)	3.5 ± 0.33 (52)
*P*	0.043	0.964	0.00007	0.018	0.317	0.185

Estimated means (±95 % CI) calculated by a general linear model within a column were compared by Dunnett *t* tests. Number of tested individuals is in brackets

^a^Means (±95 % CI) calculated from original data but statistical analysis was carried out following transformation, means were compared by *t* test

^b^Lifetime fecundity divided by oviposition period

Male longevity was unaffected by treatment (*F*(1, 53) = 0.002; *p* = 0.964) and maternal lineage (*F*(19, 53) = 0.919; *p* = 0.564) (Table 4). In addition, the survival distributions of virgin and mated males were not significantly different (Fig. 3b; log-rank test: *χ*^2^_1_ = 0.48; *p* = 0.489).

### Effects of multiple matings and male harassment on fitness of females

The cumulated number of male companions a given female was housed with had no effect on the response variables (Wilks’ Λ = 0.304; *F*(20, 64) = 1.379; *p* < 0.166; partial η^2^ = 0.257; observed power = 0.708). The model effects in the MANOVA were reported above. The follow-up Dunnett *t* tests revealed significant effects of treatment on three response variables. Specifically, although the presence of companion males had no significant effect on the longevity and daily fecundity of mated females (Table 5), mated females with a male companion had a significantly longer preoviposition period and shorter oviposition period compared to mated females housed individually (Table 5). Consequently, females with a male companion had significantly lower total fecundity (Table 5). Although the univariate test revealed a significant effect of maternal lineage (*F*(18, 69) = 2.202; *p* = 0.010; partial η^2^ = 0.365; observed power = 0.970) on the overall sex ratio of the progeny, no significant effect of treatment (*F*(1, 69) = 2.379; *p* = 0.128) was found. The overall sex ratio in the progeny produced by mated females housed alone and females with a male companion was not statistically different (Table 5). Survivorship of mated females was affected when males were kept with females. The survival curve of females housed with a single male were significantly lower than that of females housed alone (Fig. 3a; log-rank test *χ*^2^_1_ = 4.00; *p* = 0.046).

**Table 5:** Longevity, preoviposition period, oviposition period, fecundity, daily fecundity, and total offspring sex ratios of mated *Thrips tabaci* with and without a male companion

	Longevity (days)	Preoviposition period^a^ (days)	Oviposition period (days)	Fecundity(eggs/female)	Daily fecundity^*b*^(eggs/female/day)	Female offspring ratio^a^ (%)
Without male	36.5 ± 4.33 (52)	5.5 ± 1.09 (52)	29.8 ± 4.29 (52)	101.1 ± 17.0 (52)	3.5 ± 0.33 (52)	75.3 ± 4.55 (50)
With male	30.7 ± 4.71 (45)	8.5 ± 1.11 (45)	20.4 ± 4.66 (45)	63.9 ± 18.5 (45)	3.1 ± 0.35 (45)	69.0 ± 6.86 (39)
*P*	0.123	0.015	0.005	0.005	0.073	0.128

Estimated means (±95 % CI) calculated by a general linear model within a column were compared by Dunnett *t* tests. Number of tested individuals is in brackets

^a^Means (±95 % CI) calculated from original data but statistical analysis was carried out following transformation, means were compared by *t* test

^b^Lifetime fecundity divided by oviposition period

## Discussion

### Multiple matings and polyandry in *T. tabaci* females

The phenomena of multiple matings by females has been reported in a wide range of animal groups (Birkhead and Møller 1998), despite presumably large fitness costs to the female. In this study, we report that *T. tabaci* females can mate multiple times, both with the same male and with different males, which demonstrates polyandry in this species. This finding is different from a similar thrips species, *Echinothrips americanus*, in which most females mate only once during their lifespan (Li et al. 2014b). The diverse mating systems in related species and broader taxa (Thornhill and Alcock 1983) raise questions about the evolution of female mating behaviors. Several potential explanations have been proposed to understand the evolution of multiple matings and polyandry from a non-genetic view (Walker 1980; Ridley 1989; Vahed 1998; Gillott 2003) and genetic view (Yasui 1998; Jennions and Petrie 2000). Because Thysanoptera have such diverse sexual reproductive behaviors, they are a suitable group of animals in which to explore the effects of multiple matings and harassment on fitness costs to females and the evolutionary implications of such behaviors.

There is well-documented evidence that repeated mating attempts by males can lead to harassment to females which is costly to females (Arnqvist and Rowe 2005). In our study, mated females were subjected to a high harassment rate by males during all the ages we tested. The average harassment rate was over 20 times per hour, much higher than that of the observed rates (below 10 times per hour) in a similar study for *Echinothrips americanus* (Li et al. 2014b). However, contrary to *E. americanus*, there was no drop in male harassment rates when *T. tabaci* females became older than 10 days. These results suggested that post-mating interactions between females and males in *T. tabaci* include re-mating and male harassment.

### Asymmetrical fitness costs of mating to females and males

Mating is generally assumed to be costly, both for males and particularly for females. Mating is costly to females in many organisms in terms of decreased lifespan and/or reproductive output (Arnqvist and Nilsson 2000; Jormalainen et al. 2001; Macke et al. 2012). Costs of mating to males in terms of reduced longevity have also been reported in many animal species (Kotiaho and Simmons 2003; Martin and Hosken 2004; Burton-Chellew et al. 2007; Li et al. 2014b). However, asymmetrical fitness costs of mating to females and males were reported in *T. tabaci*, where mating is costly to females, but no fitness costs were found in males. In females, mating is costly in terms of reducing longevity and delaying the onset of reproduction. Mated females have a shorter lifespan and a significantly steeper decline in their survival curve than virgin females despite producing equal number of eggs. By contrast, in males, both the longevity and the survivorship curves were similar in mated males compared to virgin males. These results agree with studies in the butterfly *Lethe Diana*Butler and moth *Mnesampela private*Guenée (Walker and Allen 2010; Takeuchi 2012).

We propose that the reduced longevity in mated females is due to the trade-off for higher investment in female than male eggs. In arrhenotokous arthropods, mated females produce both female and male eggs, while virgin females only produce male eggs. In spider mites, it has been reported that mated females produce larger eggs than virgin females (Macke et al. 2012) and female eggs are larger than male eggs (Macke et al. 2011). Although the fecundity of mated females is about equal to that of virgin females in *T. tabaci*, their overall cost of investment in their progeny is still greater if male eggs have a significantly lower investment cost than female eggs, similar to spider mites (Macke et al. 2012), which may be responsible for their shorter longevity through a physiological trade-off.

### Continual exposures to males entail a cost for mated females

Results from our study indicated that multiple matings are costly to *T. tabaci* females in terms of quantitative reproductive output. Mated females kept alone had similar longevity to mated females with a male companion throughout her lifetime but higher reproductive output. A similar study reported reduced longevity of females from continuous exposure to males in the fly *Drosophila simulans*Sturtevant (Taylor et al. 2008); in contrast to this report, the fitness cost of polyandry on *T. tabaci* females was only detected by the survival analysis. Notably, continuous exposure to a male dramatically delayed the onset of reproduction and shortened the oviposition period. Male harassment might be responsible for the negatively affected survivorship and the decrease in overall fecundity, as reported in beetles and bees (Gay et al. 2009; Rossi et al. 2010). Repeated harassment by males could disturb feeding and lead to lower egg production of females. In addition, any energy expenditure by female resistance to male harassment could reduce the total investment females otherwise could spare for reproduction (Watson et al. 1998).

### The delay of the onset of oviposition because of mating, multiple matings, and male harassment

In our study, multiple matings and/or the repeated disturbance from a high rate of male harassment could delay the onset of oviposition. It has been reported that receiving the beneficial seminal accessory fluid transferred by males during mating was necessary to initiate oviposition (Barth and Lester 1973; Leopold 1976). A shorter pre-oviposition period in mated females compared to virgin females has been reported in many species (Bergh et al. 1992; Spencer and Miller 2002; Zhao and Zhu 2011; Li et al. 2012; Varikou et al. 2012). However, there are few reports about an increased pre-oviposition period in females after mating. The delay of reproduction might be due to the costly mating behavior itself or there might be some seminal accessory fluid compounds that could delay the initiation of oviposition. Continual male harassment in the first period of an adult female’s life could hinder the female’s preparation for the onset of egg production.

Sperm competition, which is a consequence of polyandry, is present in some species (Simmons 2001). The delayed oviposition might be a mechanism of mate choice. Delaying oviposition could increase the potential opportunities for sperm competition and enable females to modify their original choice of a mate by preferentially using the sperm from the most preferred male, as suggested for vertebrates (Birkhead and Moller 1993) and many other organisms including insects (Eberhard 1996; Simmons 2001). In addition, adjusting the time of oviposition might result from intersexual conflicts during post-mating interactions. In this study, the daily fecundity seemed to be unaffected by high harassment rate, and the cause of lower overall fecundity was a shortened oviposition period following a much longer preoviposition period in the continuous presence of a mate. We think that females responding to the possibility of multiple matings with the delay of oviposition are under positive selection if the possible benefit of increasing genetic diversity in progeny outweighs the cost of delayed and reduced fecundity. In other words, we think females under such conditions might be maximizing their quantitative reproductive output. This leads us to the question whether *T. tabaci* females can distinguish a previous mate from a new one and respond to multiple matings accordingly, but this remains to be examined. A similar behavior was reported for the hide beetle, *Dermestes maculates*DeGeer (Archer and Elgar 1999), and the Mediterranean flour moth, *Ephestia kuehniella*Zeller (Xu and Wang 2009): polyandrous females did not start laying eggs until they mated with several different males. The delayed oviposition could also be due to the cost of male harassment. Resistance to male harassment requires time and energy by females which would delay egg maturation. In addition, a high rate of male harassment toward females might seriously disrupt the initiation of egg laying. It is currently unclear which of the above mentioned factors cause the delay in oviposition in mated *T. tabaci*.

### Why do females mate multiple times?

The conflicts between the occurrence of multiple matings and the cost of sexual activities in this species raise the question of why females re-mate. One possible explanation for multiple matings is that, although there is no fitness benefit, females may use the sperm from different males to fertilize the eggs which could increase their offspring’s genetic diversity (Jennions and Petrie 2000). Another explanation is that females re-mate just because the avoidance of re-mating may be more costly than the cost of mating (Gavrilets et al. 2001). Mating is reported to be costly to females, and females may be reluctant to re-mate (Kokko et al. 2003). However, the avoidance of re-mating has also been reported to be costly for females (Rowe et al. 1994; Watson et al. 1998). If male harassment rates are high, resistance may be even more costly than mating itself (Rowe et al. 1994). Thus, females might accept re-mating simply to minimize the costs imposed by harassing males.

In conclusion, we found *T. tabaci* females mated multiple times and were subjected to high rates of male harassment during their lifetime. However, mating was costly to females in terms of reducing longevity and delaying the initiation of egg laying, although mating did not affect the survivorship and longevity of males. Furthermore, continual exposure to males also exerted fitness costs to mated females in terms of delaying egg production and reducing fecundity. These costs might result from multiple matings and/or a high rate of male harassment. Our results raise questions about why females in this species mate multiple times and what mechanisms cause the delay in oviposition that results from mating and exposure to males. Furthermore, we suggest that our findings in *T. tabaci* not only raise questions about the evolution of multiple matings and polyandry in this species, but that such phenomena may occur in other animal species and influence the evolution of their mating systems.

## References

[CR1] Archer MS, Elgar MA (1999). Female preference for multiple partners: sperm competition in the hide beetle, *Dermestes maculatus* (DeGeer). Anim Behav.

[CR2] Arnqvist G, Nilsson T (2000). The evolution of polyandry: multiple mating and female fitness in insects. Anim Behav.

[CR3] Arnqvist G, Rowe L (2005). Sexual conflict.

[CR4] Barth RH, Lester LJ (1973). Neuro-hormonal control of sexual behavior in insects. Annu Rev Entomol.

[CR5] Bergh JC, Harris MO, Rose S (1992). Factors inducing mated behavior in female Hessian flies (Diptera: Cecidomyiidae). Ann Entomol Soc Am.

[CR6] Birkhead TR, Moller AP (1993). Sexual selection and the temporal separation of reproductive events: sperm storage data from reptiles, birds and mammals. Biol J Linn Soc.

[CR7] Birkhead TR, Møller AP (1998). Sperm competition and sexual selection.

[CR8] Blanckenhorn WU, Hosken DJ, Martin OY, Reim C, Teuschl Y, Ward PI (2002). The costs of copulating in the dung fly Sepsis cynipsea. Behav Ecol.

[CR9] Brunner PC, Chatzivassiliou EK, Katis NI, Frey JE (2004). Host-associated genetic differentiation in *Thrips tabaci* (Insecta; Thysanoptera), as determined from mtDNA sequence data. Heredity.

[CR10] Burton-Chellew MN, Sykes EM, Patterson S, Shuker DM, West SA (2007). The cost of mating and the relationship between body size and fitness in males of the parasitoid wasp Nasonia vitripennis. Evol Ecol Res.

[CR11] Chapman T, Liddle LF, Kalb JM, Wolfner MF, Partridge L (1995). Cost of mating in Drosophila melanogaster females is mediated by male accessory gland products. Nature.

[CR12] Chen PS (1984). The functional-morphology and biochemistry of insect male accessory glands and their secretions. Annu Rev Entomol.

[CR13] Chilvers BL, Robertson BC, Wilkinson IS, Duignan PJ, Gemmell NJ (2005). Male harassment of female New Zealand sea lions, *Phocarctos hookeri*: mortality, injury, and harassment avoidance. Can J Zool-Rev Can Zool.

[CR14] Cluttonbrock TH, Parker GA (1995). Sexual coercion in animal societies. Anim Behav.

[CR15] Crespi BJ (1986). Territoriality and fighting in a colonial thrips, *Hoplothrips pedicularius*, and sexual dimorphism in Thysanoptera. Ecol Entomol.

[CR16] Crespi BJ (1988). Alternative male mating tactics in a thrips: effects of sex ratio variation and body size. Am Midl Nat.

[CR17] Crespi BJ (1988). Risks and benefits of lethal male fighting in the colonial, polygynous thrips *Hoplothrips karnyi* (Insecta: Thysanoptera). Behav Ecol Sociobiol.

[CR18] Crudgington HS, Siva-Jothy MT (2000). Genital damage, kicking and early death—the battle of the sexes takes a sinister turn in the bean weevil. Nature.

[CR19] Daly M (1978). Cost of mating. Am Nat.

[CR20] Diaz-Montano J, Fuchs M, Nault BA, Fail J, Shelton AM (2011). Onion thrips (Thysanoptera: Thripidae): a global pest of increasing concern in onion. J Econ Entomol.

[CR21] Eberhard WG (1996). Female control: sexual selection by cryptic female choice.

[CR22] Eberhard WG, Cordero C (1995). Sexual selection by cryptic female choice on male seminal products—a new bridge between sexual selection and reproductive physiology. Trends Ecol Evol.

[CR23] Franklin AM, Squires ZE, Stuart-Fox D (2012). The energetic cost of mating in a promiscuous cephalopod. Biol Lett.

[CR24] Gavrilets S, Arnqvist G, Friberg U (2001). The evolution of female mate choice by sexual conflict. Proc R Soc B-Biol Sci.

[CR25] Gay L, Eady PE, Vasudev R, Hosken DJ, Tregenza T (2009). Costly sexual harassment in a beetle. Physiol Entomol.

[CR26] Gilbert JDJ, Simpson SJ (2013). Natural history and behaviour of *Dunatothrips aneurae* Mound (Thysanoptera: Phlaeothripidae), a phyllode-gluing thrips with facultative pleometrosis. Biol J Linn Soc.

[CR27] Gillott C (2003). Male accessory gland secretions: modulators of female reproductive physiology and behavior. Annu Rev Entomol.

[CR28] Gotoh T, Tsuchiya A (2008). Effect of multiple mating on reproduction and longevity of the phytoseiid mite Neoseiulus californicus. Exp Appl Acarol.

[CR29] Helinski MEH, Harrington LC (2012). The role of male harassment on female fitness for the dengue vector mosquito Aedes aegypti. Behav Ecol Sociobiol.

[CR30] Jennions MD, Petrie M (2000). Why do females mate multiply? A review of the genetic benefits. Biol Rev.

[CR31] Ji J, Zhang ZQ, Zhang YX, Chen X, Lin JZ (2007). Effects of mating rates on oviposition, sex ratio and longevity in a predatory mite *Neoseiulus cucumeris* (Acari : Phytoseiidae). Exp Appl Acarol.

[CR32] Johnson SL, Brockmann HJ (2010). Costs of multiple mates: an experimental study in horseshoe crabs. Anim Behav.

[CR33] Jormalainen V, Merilaita S, Riihimaki J (2001). Costs of intersexual conflict in the isopod Idotea baltica. J Evol Biol.

[CR34] Kokko H, Brooks R, Jennions MD, Morley J (2003). The evolution of mate choice and mating biases. Proc R Soc Lond Ser B-Biol Sci.

[CR35] Kotiaho JS, Simmons LW (2003). Longevity cost of reproduction for males but no longevity cost of mating or courtship for females in the male-dimorphic dung beetle *Onthophagus binodis*. J Insect Physiol.

[CR36] Kotiaho J, Alatalo RV, Mappes J, Parri S, Rivero A (1998). Male mating success and risk of predation in a wolf spider: a balance between sexual and natural selection?. J Anim Ecol.

[CR37] Kotiaho JS, Alatalo RV, Mappes J, Nielsen MG, Parri S, Rivero A (1998). Energetic costs of size and sexual signalling in a wolf spider. Proc R Soc B-Biol Sci.

[CR38] Leboeuf BJ, Mesnick S (1991). Sexual behavior of male northern elephant seals. 1. Lethal injuries to adult females. Behaviour.

[CR39] Leopold RA (1976). The role of male accessory glands in insect reproduction. Annu Rev Entomol.

[CR40] Lewis T (1973). Thrips: their biology, ecology and economic importance.

[CR41] Li X-W, Zhang X-C, Jiang H-X, Feng J-N (2012). Comparisons of developmental and reproductive biology between parthenogenetic and sexual *Echinothrips americanus* (Thysanoptera: Thripidae). Environ Entomol.

[CR42] Li X-W, Fail J, Wang P, Feng J-N, Shelton AM (2014). Performance of arrhenotokous and thelytokous *Thrips tabaci* (Thysanoptera: Thripidae) on onion and cabbage and its implications on evolution and pest management. J Econ Entomol.

[CR43] Li XW, Jiang HX, Zhang XC, Shelton AM, Feng JN (2014). Post-mating interactions and their effects on fitness of female and male *Echinothrips americanus* (Thysanoptera: Thripidae), a new insect pest in China. PLoS ONE.

[CR44] Macke E, Magalhaes S, Khan HDT, Luciano A, Frantz A, Facon B, Olivieri I (2011). Sex allocation in haplodiploids is mediated by egg size: evidence in the spider mite Tetranychus urticae. Koch Proc R Soc B-Biol Sci.

[CR45] Macke E, Magalhães S, Khanh HD-T, Frantz A, Facon B, Olivieri I (2012). Mating modifies female life history in a haplodiploid spider mite. Am Nat.

[CR46] Magnhagen C (1991). Predation risk as a cost of reproduction. Trends Ecol Evol.

[CR47] Martin OY, Hosken DJ (2004). Copulation reduces male but not female longevity in *Saltella sphondylli* (Diptera: Sepsidae). J Evol Biol.

[CR48] Oku K (2010). Males of the two-spotted spider mite attempt to copulate with mated females: effects of double mating on fitness of either sex. Exp Appl Acarol.

[CR49] Parker GA, Blum MS, Blum NA (1979). Sexual selection and sexual confilict. Sexual selection and reproductive competition in insects.

[CR50] Plath M (2008). Male mating behavior and costs of sexual harassment for females in cavernicolous and extremophile populations of Atlantic mollies (Poecilia mexicana). Behaviour.

[CR51] Ridley M (1989). The timing qnd frequency of mating in insects. Anim Behav.

[CR52] Rolff J, Siva-Jothy MT (2002). Copulation corrupts immunity: a mechanism for a cost of mating in insects. Proc Natl Acad Sci U S A.

[CR53] Ronkainen K, Kaitala A, Kivela SM (2010). Polyandry, multiple mating, and female fitness in a water strider *Aquarius paludum*. Behav Ecol Sociobiol.

[CR54] Rossi BH, Nonacs P, Pitts-Singer TL (2010). Sexual harassment by males reduces female fecundity in the alfalfa leafcutting bee, *Megachile rotundata*. Anim Behav.

[CR55] Rowe L, Arnqvist G, Sih A, Krupa J (1994). Sexual conflict and the evolutionary ecology of mating patterns: water striders as a model system. Trends Ecol Evol.

[CR56] Simmons LW (2001). Sperm competition and its evolutionary consequences in the insects.

[CR57] Sirot LK, Brockmann HJ (2001). Costs of sexual interactions to females in Rambur's forktail damselfly, *Ischnura ramburi* (Zygoptera : Coenagrionidae). Anim Behav.

[CR58] Spencer JL, Miller JR (2002). Lifetime ovipositional patterns of mated and virgin onion flies, *Delia antiqua* (Diptera : Anthomyiidae). J Insect Physiol.

[CR59] Tabachnick BG, Fidell LS (2007). Using multivariate statistics.

[CR60] Takeuchi T (2012). Cost of reproduction in males of a satyrine butterfly *Lethe diana*. Physiol Entomol.

[CR61] Taylor ML, Wigmore C, Hodgson DJ, Wedell N, Hosken DJ (2008). Multiple mating increases female fitness in Drosophila simulans. Anim Behav.

[CR62] Terry I, Schneider M (1993). Copulatory behaviour and mating frequency of the western flower thrips, *Frankliniella occidentalis* (Insecta: Thysanoptera). Zool (J Pure Appl Zool).

[CR63] Thornhill R (1976). Sexual selection and paternal investment in insects. Am Nat.

[CR64] Thornhill R, Alcock J (1983). The evolution of insect mating systems. The evolution of insect mating systems.

[CR65] Toda S, Murai T (2007). Phylogenetic analysis based on mitochondrial COI gene sequences in *Thrips tabaci* Lindeman (Thysanoptera: Thripidae) in relation to reproductive forms and geographic distribution. Appl Entomol Zool.

[CR66] Vahed K (1998). The function of nuptial feeding in insects: review of empirical studies. Biol Rev Camb Philos Soc.

[CR67] Varadarasan S, Ananthakrishnan TN (1982). Biological studies on some gall thrips. Proc Indian Natl Acad Sci.

[CR68] Varikou K, Birouraki A, Tsitsipis I, Sergentani CHR (2012). Effect of temperature on the fecundity of *Pezothrips kellyanus* (Thysanoptera: Thripidae). Ann Entomol Soc Am.

[CR69] Walker WF (1980). Sperm utilization strategies in nonsocial insects. Am Nat.

[CR70] Walker PW, Allen GR (2010). Mating frequency and reproductive success in an income breeding moth, *Mnesampela privata*. Entomol Exp Appl.

[CR71] Watson PJ, Arnqvist G, Stallmann RR (1998). Sexual conflict and the energetic costs of mating and mate choice in water striders. Am Nat.

[CR72] Wing SR (1988). Cost of mating for female insects: risk of predation in *Photinus collustrans* (Coleoptera: Lampyridae). Am Nat.

[CR73] Xu J, Wang Q (2009). A polyandrous female moth discriminates against previous mates to gain genetic diversity. Anim Behav.

[CR74] Yasui Y (1998). The 'genetic benefits' of female multiple mating reconsidered. Trends Ecol Evol.

[CR75] Zhao LQ, Zhu DH (2011). Effect of mating status on the fecundity of a cricket, *Teleogryllus emma*. Insect Sci.

